# Relationship Between Perceived and Received Social Support in Family Caregivers: A Systematic Review with Meta-Analysis

**DOI:** 10.3390/nursrep14040252

**Published:** 2024-11-12

**Authors:** Belén Gutiérrez-Sánchez, Catalina López-Martínez, Henrique da-Silva-Domingues, Rafael del-Pino-Casado

**Affiliations:** Nursing Department, University of Jaen, 23071 Jaén, Spain; bgutierr@ujaen.es (B.G.-S.); hda@ujaen.es (H.d.-S.-D.); rdelpino@ujaen.es (R.d.-P.-C.)

**Keywords:** perceived social support, received social support, informal caregivers, meta-analysis

## Abstract

**Background:** The care of dependent people is eminently family-oriented, and often, there is a high level of dedication to this family care. Constant and continuous care leads to a series of negative psychological consequences. Social support has been related to improved mental health in family caregivers. We found heterogeneous results regarding the relationship between the types of social support received and the perceived level of support. In addition, to our knowledge, no reviews analyse this relationship among family caregivers. **Objectives**: Therefore, we objective to systematically synthesise the relationships between perceived and received social support in informal caregivers. **Methodology**: We have carried out a quantitative systematic review with a meta-analysis, registered in PROSPERO (id: CRD42023470047); the systematic search was carried out in the following databases: PubMed, CINAHL, PsycINFO, and Scopus, until November 2023. After the selection and review of the results, twelve studies were obtained, two of which were eliminated due to a high risk of classification bias. **Results**: Regarding the results, a medium-size positive statistical association was found (r = 0.43). The results were consistent, accurate, and robust. The Trim and Fill test showed a variation of 7%. Subgroup analysis indicated no differences in the age group of the people cared for (adults or children), selection bias, and confounding bias. **Conclusions**: In conclusions, perceived social support is related to more social support received by family caregivers.

## 1. Introduction

Cohen et al. describe social support [1] as “social resources that people perceive as available or that are actually provided by non-professionals in the context of both formal support groups and informal helping relationships”.

Types of social support vary depending on the source (family, friends, neighbours), the type of transfer that is carried out (emotional, instrumental, informational) [2], and the type of support (received or perceived) [3]. Perceived social support assesses subjective aspects, satisfaction, and perception of the availability of support, while social support received is associated with transaction frequency [3].

Numerous studies link social support to better mental or psychological health [4,5,6]. Thus, those who have a higher level of social support may have better levels of physical or psychological health than those who are more socially isolated [3]. We found reviews that analyse the relationship between social support and some of the most frequent psychological consequences in family caregivers such as anxiety [7], depression [8], or subjective overload [9].

Social support has been specifically analysed in different populations, with family caregivers being one of the most studied because the context of family care offers good conditions for analysing the relationships between social support and emotional consequences [10]. Perceived social support provides the requirements or resources necessary to assess the problem, while received social support or network size has an impact on stress relief and acts on the perceived importance of the problem either through avoidance or through healthy behaviours that help the family caregiver, in this case, to cope with that need [9].

The care of dependent people is eminently family-oriented, and a high level of dedication to this family care is common [11]. Permanent care for a dependent family member has significant emotional repercussions [12,13,14]. Among these problems, subjective overload, depression, and anxiety stand out [12,13,14]. An important aspect that has been studied in the literature on caregivers has been the relationships between social support, overload, anxiety, and depression [7,8,9].

Lazarus and Folkman’s theory on stress-buffering [15] expresses how social support acts as a palliative resource against the physical and mental effects produced by stress. This theory supports the connection between received and perceived support, which should be relatively strong and therefore positive perceptions of support, and the reception of support should have stress-mitigating results, especially in family caregivers of dependent persons [16,17,18].

With respect to the relationship between received and perceived support, we find polar opposites. Thus, some studies indicate that both supports are related, with perceived support arising from that received [19]. We also have studies that indicate that both supports are independent, as the correlation between them is weak [20].

It should be noted that very few studies look at the effects of both supports together [20]. Different authors point out the positive relationship between perceived social support with respect to health [20,21,22]. However, the relationship between social support received and health is very ambiguous. This may contribute to more studies focusing on perceived support [20].

Implementing interventions to improve social support is necessary to prevent or improve the burden of informal caregivers [9]. However, we do not know what relationship there is between both types of support and thus need to direct the interventions to a more specific point since we have found original studies on the subject but not systematic reviews. Thus, this study aims to systematically synthesise the relationships between perceived and received social support in informal caregivers.

## 2. Materials and Methods

### 2.1. Design

We developed a quantitative systematic review with meta-analysis, following the suggestions of PRISMA [23] and the Cochrane Handbook [24] in order to achieve our established objective.

This review has been registered in PROSPERO [25] with Id no. CRD42023470047.

### 2.2. Search Strategy

We conducted a systematic search using the following databases: PubMed, CINAHL, PsycINFO, and Scopus, up to October 2024. Table S1 shows the search strings used in the different databases.

### 2.3. Eligibility Criteria

The inclusion criteria established were (1) original studies, (2) with reports on the relationship between perceived support and support received, (3) informal caregivers (4) of dependent people (with no age limit), (5) to present statistical data corresponding to the magnitude of association between perceived and received social support.

### 2.4. Data Extraction

For data extraction, two review authors (BGS and RdPC) extracted data from articles independently using a standardised form.

The following were collected: author and year, type of design, sample size, age group of caregivers (children or adults), mean age, length of care, percentage of female caregivers, percentage of caregiver spouses, scale of perceived social support, scale of social support received, cause of dependence of the people cared for, and size of association.

### 2.5. Quality Assessment of Included Studies

To determine the methodological quality, the following criteria recommended by Viswanathan et al. [26] and Boyle [27] were assessed: selection bias, which was considered controlled when probabilistic samples were used; classification bias or control of the validity and reliability of the instruments used; and confounding bias, which was assessed as controlled when confounding variables (support network size, age and sex of informal caregivers) were controlled. We emphasise that control of classification bias was mandatory for the inclusion of one study in the review.

We consider that there is control of confounding bias for any of the aforementioned variables when there are allocation mechanisms that guarantee the formation of groups that are comparable according to those variables (e.g., through stratification, matching, or propensity scores) or when statistical adjustment mechanisms are used for these variables (e.g., stratified or multivariate analysis) [26]. In the statistical adjustment, we consider that there is no confounding bias when the variation between the size of the crude effect and the adjusted effect is less than 10% [28]

The review of the quality criteria of the different studies was carried out by two authors (BGS and RdPC) independently.

### 2.6. Assessing the Quality of the Evidence

Following the recommendations of the System for the Evaluation, Development and Evaluation of Recommendations (GRADE) [29], we assessed the quality of the evidence using the methodological quality of the included studies (described in the previous paragraph), inconsistency (degree of heterogeneity), imprecision, and publication bias.

The evaluation of the heterogeneity of the results in the different selected studies was carried out via several methods (see Section 2.7). To measure imprecision, two criteria were taken into account: the number of studies included (small: less than 5 studies, medium: between 5 and 10 studies, and adequate: more than 10 studies) and the sample size (small: less than 100 participants, medium: from 100 to 300 participants, and large: more than 300 participants). Finally, to evaluate the publication bias, the appraisal of the funnel plot and several statistical tests explained in the Section 2.7 were analysed.

### 2.7. Analysis

A random effects model was conducted following the recommendations of Cooper et al. [30] in order to generalise the results obtained to any population of family caregivers. All analyses described below were performed using the Comprehensive Meta-Analysis 3.3 program.

Regarding the analysis of heterogeneity, we used the Q test (alpha value: 0.1) to test the similarity of effect sizes between the included studies and the Higgins inconsistency score (I^2^) [31] to establish the proportion of observed variability between studies that was not due to chance. Values of Higgins I^2^ statistics range from 0% (absence of heterogeneity) to 100% (maximum level of heterogeneity), with the following cut-off points: 25% (low heterogeneity), 50% (moderate), 75% (high) [31].

Several methods were used to assess publication bias based on the recommendations established by Guyatt et al. [32]. The methods used were inspection of the degree of asymmetry of the funnel plot, the test of Egger et al. [33], and the Trim and Fill method [34].

The Egger test evaluates the symmetry of the funnel plot testing the null hypothesis that the slope in the regression of precision on effect size is zero, with an alpha value of 0.1 [33]. The Trim and Fill method estimates the combined effect in a scenario of absence of publication bias [34].

To investigate the robustness of our findings, we performed leave-one-out meta-analyses, excluding one study at each analysis. In addition, subgroup analyses were carried out to study robustness and possible moderating effects in the combined effect. We analysed the moderating effect of age group (adults vs. children) and methodological quality criteria (absence of selection bias and confounding bias).

## 3. Results

### 3.1. Description of the Search Results

**Figure 1:**
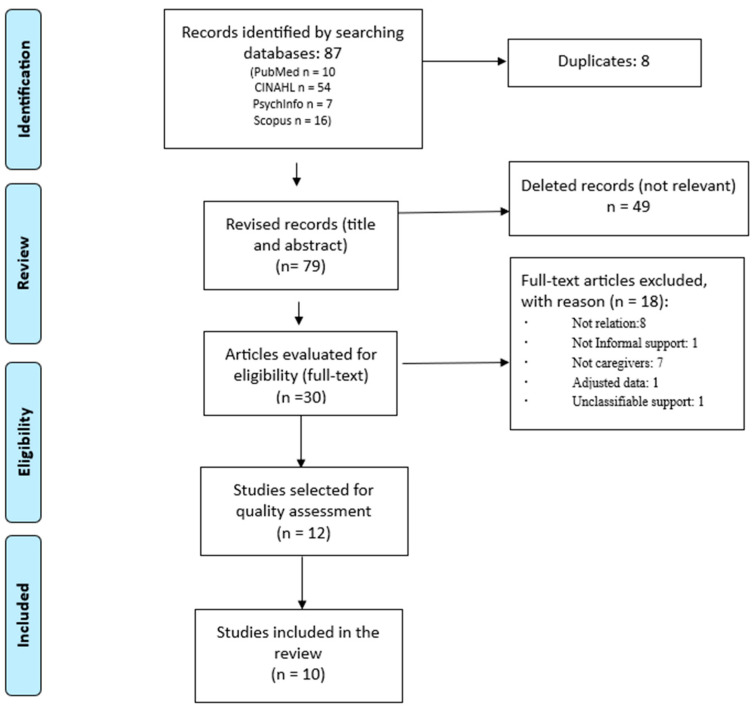
Flow diagram of the review on the relationship between perceived and received social support.

We obtained a total of eighty-seven results from the different databases, of which we eliminated eight studies because they were duplicates. Of the remaining 79, 49 were eliminated because they were not relevant to the topic studied, leaving 30 studies to be reviewed in full text. Of these thirtry, eighteen were eliminated according to the different inclusion criteria (eight for not relating the support received and received, one for analysing formal support, seven for studying populations other than those of caregivers, one for providing adjusted data, and one for containing unclassifiable support). The quality of the twelve selected studies was reviewed, eliminating two due to classification bias, leaving a final sample of ten studies (Figure 1).

### 3.2. Description of the Characteristics of the Studies

As can be seen in Table 1, the ten studies included were cross-sectional descriptive studies, one of them being repeated measures but with cross-sectional correlations. A total of 70% of the studies (*n* = 7) were conducted in the United States. A total of 50% of the samples were obtained by non-probability sampling. The total sample was 2142 people, with a range of between 17 and 637 caregivers. The year of publication ranged from 1994 to 2023. With respect to care recipients, those in half of the studies (*n* = 5) were adults. The most frequent causes of dependence were dementia (*n* = 3) and autism (*n* = 2).

**Table 1:** Characteristics of the included studies.

Study (Author-Year) Country	N	Mean Age (SD) and Range	Percentage of Female (%)	Design	Recipients of Care	Measure of Perceived Social Support *	Measure of Received Social Support **
Benson 2009 [35] USA	96	41.9 (5.04) 30–55	100	Repeated measures with cross-sectional correlations	Autism Children	MSPSS	ISSB
Burton 2008 [36] USA	50	72.8 (10.2) NA	80	Cross-sectional	Cancer and dementia Adult	KB-C	KB-C
Dolcini-Catania 2021 [37] USA	367	NA	95	Cross-sectional	Behavioral problems Children	GLS	GLS
Falzarano 2022 [38] USA	243	60.97 (11.91) 20–95	NA	Cross-sectional	Frail older people Adult	KB-C	ISSB
Kaul 2003 [39] USA	60	28 (NA) NA	100	Cross-sectional	Heart problems Children	SPS	ISSB
Losada 2010 [40] Spain	334	58.6 (12.9) 28–85	77.8	Cross-sectional	Dementia Adults	PSQ	PSQ
Mariñez-Lora 2021 [20] USA	89	32 (7.9) 20–53	100	Cross-sectional	Mental illness Children	NSSQ	ISSQ
Robinson 2020 [41] Canada	249	44 (6.2) 27–64	95.6	Cross-sectional	Autism Children	SPS	ISSB
Robison 1994 [42] Georgia	17	63.5 (NA) 52–80	100	Cross-sectional	Dementia Adults	GSS	SNL-A
Xu 2017 [43] USA	637	60.5 (13.3) 23–90	82.9	Cross-sectional	Dementia Adults	LSN	Ad-hoc

* MSPSS: Multidimensional Scale of Perceived Social Support, GLS: General Life Satisfaction, SPS: Social Provisions Scale, PSQ: Psychosocial Support Questionnaire, NSSQ: Norbeck Social Support Questionnaire at baseline, GSS: Global Satisfaction Scale. ** Inventory of Socially Supportive Behaviours, PSQ: Psychosocial Support Questionnaire, ISSQ: Inventory of Socially Supportive Behaviours, GLS: General Life Satisfaction, SNL-A: Social Network List (current contact) (For more Information see Table S2).

### 3.3. Description of the Quality of the Studies

The results of the quality assessment of the ten included studies can be seen in Table 2. Eight present studies had non-probability samples and two studies controlled for confounding variables.

### 3.4. Description of Meta-Analysis Results

**Figure 2:**
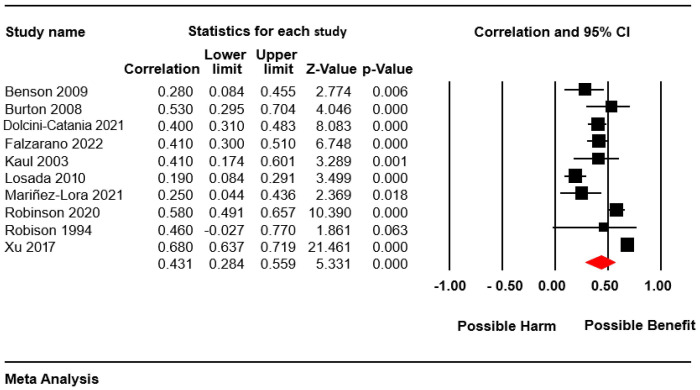
Forest plot [20,35,36,37,38,39,40,41,42,43].

Ten studies reported the relationship between perceived and received social support, showing a positive statistical association with a median effect size (r = 0.43, confidence interval [CI] = 0.28; 0.56, N = 2142; Mean N = 214.2; Figure 2). All of the studies reported positive correlations, although in one study, the correlation was not statistically significant.

We reached an absence of heterogeneity, so the results were consistent (*p* value for the Q test: 0.84; I^2^ = 0.0), showing adequate precision (10 studies with an average of 214.2 participants per study) and adequate robustness (the leave-one-study meta-analysis showed a maximum variation of 9.9% with respect to the combined effect).

**Figure 3:**
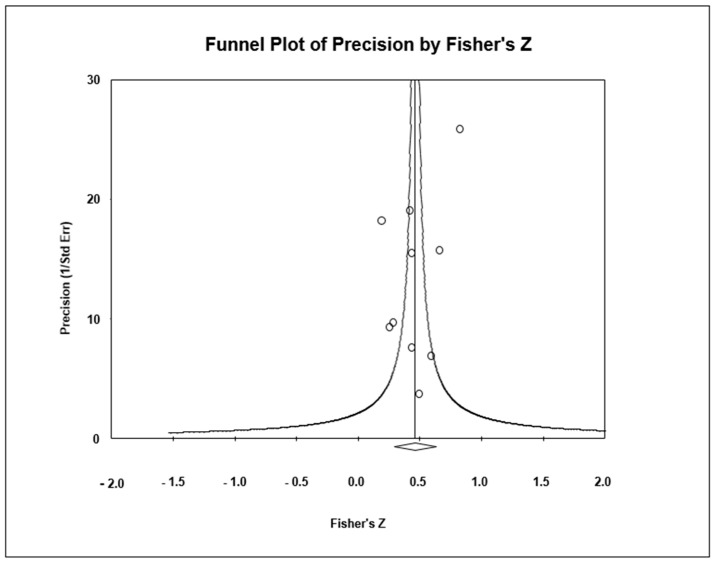
Funnel plot for perceived social support and received social support.

The funnel plot (Figure 3) appeared somewhat asymmetrical, but no greater magnitude of association was observed in the small studies or studies with an extreme magnitude of association. In addition, in the Egger test, a value of *p* = 0.81 was obtained. The Trim and Fill test indicates a value of 0.46 (7% variation from the actual combined effect). Thus, there is a risk of publication bias, but such bias could have a very small impact on the outcome of the meta-analysis.

The subgroup analysis indicated no statistically significant difference in effect size when comparing the age group of people cared for (adults: r = 0.47; 95% CI = 0.19, 0.68; five studies; children: r = 0.4; 95% CI = 0.27, 0.52; five studies). With regard to quality criteria, no differences were found according to the type of sampling (studies with non-probability samples: r = 0.49; 95% CI = 0.26, 0.67; five studies; studies with probability samples: r = 0.38; 95% CI = 0.23; 0.51; five studies), and between those studies that controlled for confounding bias (r = 0.43; 95% CI = 0.33; 0.52; two studies) and those that did not control (r = 0.5; 95% CI = 0.46; 0.53; eight studies).

## 4. Discussion

To our knowledge, our review is the first to study the relationship between perceived social support and the social support received by family caregivers. We found that higher levels of perceived social support are associated with higher levels of social support received from family caregivers.

The results obtained indicated a value of the size of the median effect and were consistent, accurate, and robust. After subgroup analysis, no differences were found according to the age group of the person cared for (adults or children) or according to quality criteria (selection bias and confounding bias).

We found similar results, but with a higher magnitude of association, in the systematic review of Haber et al. [44], which analyses the relationship between social support received (measured by the Inventory of Socially Supportive Behaviours scale (ISSB) [45] and perceived social support (measured by the Interpersonal Support Evaluation List (ISEL) and Social Support Questionnaire scales (SSQ) [46]) in the adult population. Another study carried out by [47] in the adult population obtained the same results—a positive statistical association between perceived social support and received social support.

According to the existing literature on family caregivers of children diagnosed with autism spectrum disorder [48], both perceived social support and received social support play a key role in reducing stress levels in this population, demonstrating that both concepts are related with values very similar to ours [40]. Such an association increases when support needs are considered, i.e., the number of times support is received when needed [3]. Social support acts as a modulating variable in negative health consequences according to the theory of the “Buffer Effect Hypothesis” [1]. In family caregivers of dependent adults, social support has been linked to depressive symptoms and anxiety, with dementia being the most common cause of dependency [7,8,9].

Perceived social support and social support received have been studied in various contexts and in relation to different negative consequences of care [7,8,9]. Evidence so far has provided mixed results regarding the relationship between perceived and received support, from studies that indicate that they act differently and that they are distinct concepts to studies that support that both are interrelated [19]. The correlation obtained between perceived and received support sustain the theory that, although they are related, they are different constructs.

Various studies have shown that both types of support are related differently to the different negative consequences of caregiving, with the possible protective effect of perceived support being greater than that received with respect to subjective overload [9], anxiety [7], and depression [8]. Our results support that both types of support are different constructs, and together with previous results that show a possible greater protective effect of perceived support, support prioritizing action on perceived social support over the support received, if necessary, when planning interventions aimed at promoting social support.

This study has limitations. Firstly, there is the low number of included studies, which is due to the scarcity of research on the subject and the establishment as a mandatory inclusion criterion of the validity and reliability of measures used to measure social support, although this last aspect increases the quality of the results. Secondly, all of the studies included in the meta-analysis have a cross-sectional descriptive design, which prevents the establishment of causal relationships. Most of the studies included in the meta-analysis have non-probabilistic samples and do not control for potential confounding variables, although in the sensitivity analyses, there were no significant differences in the combined effect according to these quality criteria. And finally, there is the heterogeneity of the instruments used to measure perceived and received social support.

## 5. Conclusions

This study provides the first quantitative synthesis of the relationship between perceived social support and social support received in family caregivers. The results obtained indicate that perceived social support is related to more social support received by family caregivers. Although both types of support are related, the magnitude of the association between them supports the argument that they are different constructs. When planning interventions to promote social support, it may be appropriate to prioritise actions aimed at perceived social support over support received, where necessary. For example, one may prioritise the promotion of group activities within the Primary Care centre, where the family caregiver tells their experience and receives advice from other people in the same situation as well as from health professionals.

Further longitudinal studies will be needed in future research to investigate the possible causal relationships between perceived social support and received social support.

## Figures and Tables

**Table 2:** Quality evaluation.

Author and Year	Selection	Classification	Confounding
Benson 2009 [35]	−	+	−
Burton 2008 [36]	−	+	+
Dolcini-Catania 2021 [37]	+	+	−
Falzarano 2022 [38]	−	+	+
Kaul 2003 [39]	−	+	−
Losada 2010 [40]	−	+	−
Mariñez-Lora 2021 [20]	+	+	−
Robinson 2020 [41]	−	+	−
Robison 1994 [42]	−	+	−
Xu 2017 [43]	−	+	−

Note: (−) Risk of bias; (+) Low risk of bias

## Data Availability

All data are available from the authors upon reasonable request.

## Supplementary Materials

The following supporting information can be downloaded at: https://www.mdpi.com/article/10.3390/nursrep14040252/s1, Table S1: Search strategy. Table S2: Information on the instruments used to measure the types of social support.

## Public Involvement Statement

There was no public involvement in any aspect of this research.

## Guidelines and Standards Statement

This manuscript was drafted against the PRISMA (An updated guideline for reporting systematic reviews [23]) for reporting systematic reviews and meta-analyses research.
