# Supplementary material for: Relationship Between Perceived and Received Social Support in Family Caregivers: A Systematic Review with Meta-Analysis
Source: Nurs Rep. 2024 Nov 12;14(4):3466–75. doi: 10.3390/nursrep14040252 (PMC11587471; doi:10.3390/nursrep14040252)
Supplement: Supplementary file 1 [file nursrep-14-00252-s001.zip › nursrep-3250212-supplementary.pdf]

Table S1: Search strategy

Data base	Chain Used	Search date	Number of results
**Pubmed**	(perceived social support[tiab] OR perceived support[tiab]) AND (received social support[tiab] OR received support[tiab]) AND (caregiver[mh] OR Caregiv*[tiab] OR Care giv*[tiab] OR Carer*[tiab])	28/10/24	10
**CINAHL**	(AB perceived social support OR AB perceived support) AND (MH Caregivers OR AB Caregiv* OR AB Care giv* OR AB Carer*) AND (AB Received social support OR AB received support)	28/10/24	54
**PsyInfo**	AB("received social support") AND (SU(Caregivers) OR AB(caregiv*) OR AB(care giv*) OR AB(carer*)) AND AB("perceived social support")	28/10/24	7
**Scopus**	(INDEXTERMS(Caregivers) OR TITLE-ABS-KEY(carer*) OR TITLE-ABS-KEY(caregiv*) OR TITLE-ABS-KEY(care giv*)) AND (TITLE-ABS-KEY("Received social support") OR TITLE-ABS-KEY("Received support")) AND (TITLE-ABS-KEY("Perceived social support") OR TITLE-ABS-KEY("Perceived support"))	28/10/24	16

Table S2: Information on the instruments used to measure the types of social support

ACRONYM	FULL NAME	TYPE OF SUPPORT	REFERENCE
**MSPSS**	Multidimensional Scale of Perceived Social Support	Perceived social support	Zimet GD, Dahlem NW, Zimet SG, Farley GK. The multidimensional scale of perceived social support. J Pers Assess 1988;52(1):30–41.
**KBC**	Krause and Borawski-Clark	Perceived and received social support	Krause N, Borawski-Clark E. Social class differences in social support among older adults. Gerontologist 1995;35(4):498–508.
**GLS**	General Life Satisfaction	Perceived and received social support	Crnic KA, Greenberg MT, Ragozin AS, Robinson NM, Basham RB. Effects of stress and social support on mothers and premature and full-term infants. Child Dev 1983:209–217.
**SPS**	Social Provisions Scale	Perceived social support	Cutrona CE, Russell DW. The provisions of social relationships and adaptation to stress. Advances in personal relationships 1987;1(1):37–67.
**PSQ**	Psychosocial Support Questionnaire	Perceived and received social support	Reig A, Ribera D, Miquel J. Psychological support and daily stress in non-institutionalized elderly. Evaluación Psicológica 1991.
**NSSQ**	Norbeck Social Support Questionnaire at baseline	Perceived social support	Norbeck JS, Lindsey AM, Carrieri VL. Further development of the Norbeck Social Support Questionnaire: Normative data and validity testing. Nurs Res 1983;32(1):4–9.
**GSS**	Global Satisfaction Scale	Perceived social support	Fiore J, Coppel DB, Becker J, Cox GB. Social support as a multifaceted concept: Examination of important dimensions for adjustment. Am J Community Psychol 1986;14(1):93.

**ISSB**	Inventory of Socially Supportive Behaviors	Received social support	Stokes JP, Wilson DG. The inventory of socially supportive behaviors: dimensionality, prediction and gender differences. Am J Community Psychol 1984;12(1):53.
**SNL-A**	Social Network list (current contact)	Received social support	Fiore J, Coppel DB, Becker J, Cox GB. Social support as a multifaceted concept: Examination of important dimensions for adjustment. Am J Community Psychol 1986;14(1):93.
**LSN**	Lubben Social Network	Received social support	Lubben JE. Assessing social networks among elderly populations. Fam Community Health 1988;11(3):42–52.
